# Supplementary material for: Optimizing brinjal (Solanum melongena L.) health and yield through bio-organic amendments against Fusarium wilt
Source: PLoS One. 2025 Oct 9;20(10):e0325345. doi: 10.1371/journal.pone.0325345 (PMC12510524; doi:10.1371/journal.pone.0325345)

S1 Appendix:

T_0 = (Control)

T_1 = Spent mushroom substrate

T_2 = Vermicompost

T_3 = Poultry manure

T_4 = Biochar

T_5 = Spent mushroom substrate + Biochar

T_6 = Spent mushroom substrate + Poultry manure

T_7 = Spent mushroom substrate + Vermicompost

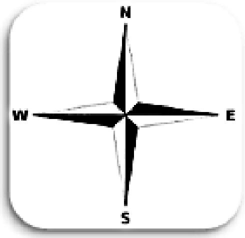

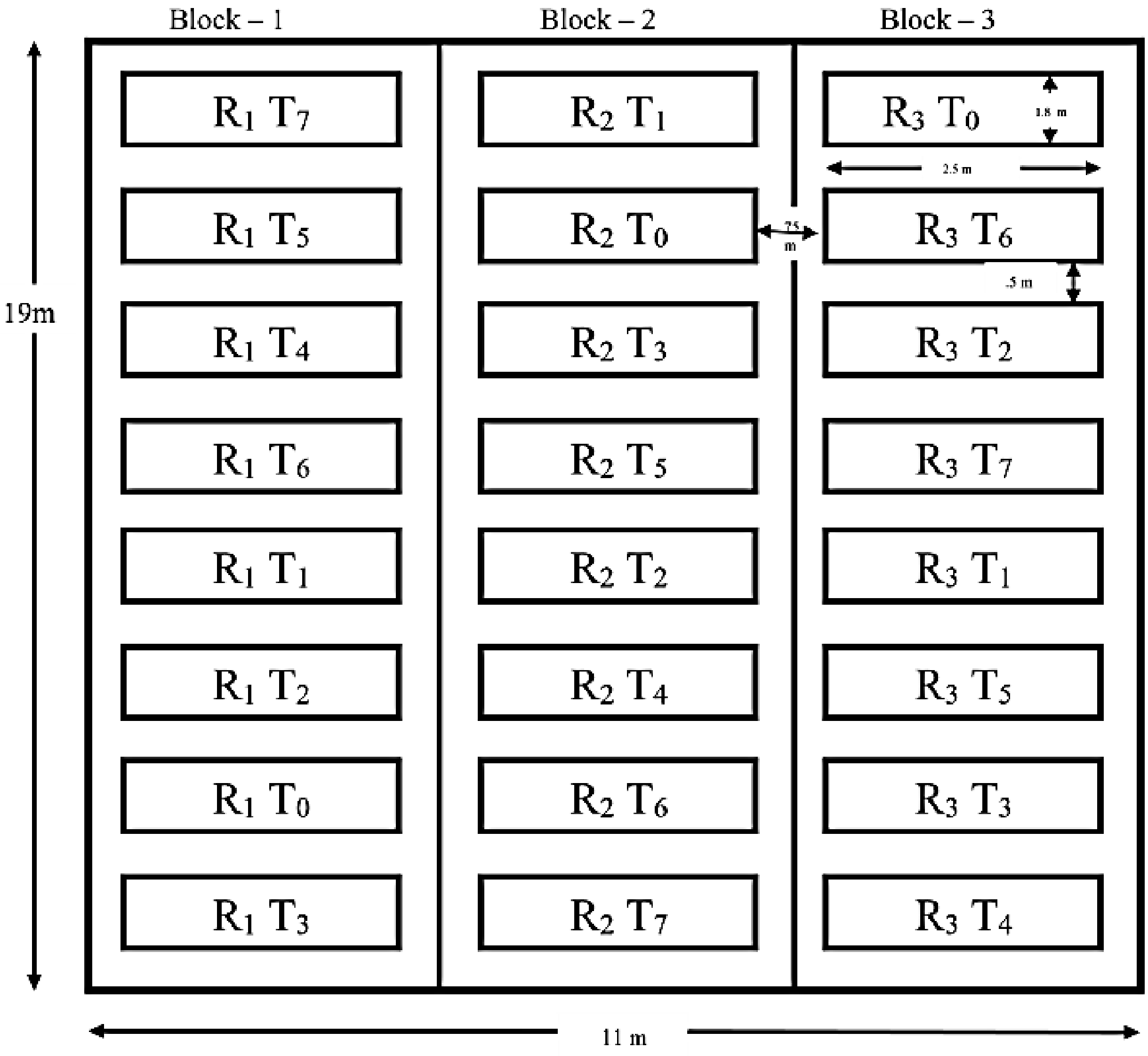

Supplement: S1 Appendix — (PDF) [file pone.0325345.s001.pdf]
