# Supplementary material for: Optimizing brinjal (Solanum melongena L.) health and yield through bio-organic amendments against Fusarium wilt
Source: PLoS One. 2025 Oct 9;20(10):e0325345. doi: 10.1371/journal.pone.0325345 (PMC12510524; doi:10.1371/journal.pone.0325345)
Supplement: Attachment — Submitted filename: Response to Reviewers.pdf [file pone.0325345.s003.pdf]

Date: 09-08-2025

Academic Editor,
PLOS ONE

Dear Ravinder Kumar,

The author would like to thank you and the reviewers for your thoughtful and constructive feedback on our manuscript [PONE-D-25-25430]. The author greatly appreciates the time and effort put into reviewing our work, and we have carefully considered each point raised. In response, the author has made the necessary revisions and clarifications throughout the manuscript to address all concerns.

Enclosed is a table detailing each comment made by the reviewers, alongside author responses and the corresponding revisions. The author believes these changes have strengthened the manuscript, and we hope that it now meets the journal's standards.

**Reviewer #1**	**Response by Author**
1. **Abstract**<br>Line 17: The authors state that the pathogen was identified by morphological and pathogenicity tests. However there are several very similar Fusarium species which can infect S. melongena. I'd suggest to do at least a PCR test for tef1 gene and then sequence the specific product to have a molecular identification of the pathogen. OR the authors could have used an already molecularly identified isolate from a culture collection.	Thank you for your valuable suggestion. We fully agree that molecular techniques such as PCR amplification and sequencing of the *tef1* gene are more precise for species-level identification, especially for closely related *Fusarium* species infecting *Solanum melongena*. However, the primary objective of this study was focused on disease management rather than taxonomic resolution. Therefore, molecular identification was not performed at the time. Additionally, as the experiment was conducted during the 2021–2022 period, the fungal cultures are no longer available for retrospective molecular analysis. In response to this limitation and to avoid over-specification, we have revised the manuscript to refer to the causal agent as *Fusarium* sp. rather than *Fusarium oxysporum* f. sp. *melongenae*.
2. It is also not clear if the experiment was done once or it was repeated and if there were repetitions of the treatment. This important information is in the Materials and Methods section, please add it to the abstract, because it makes the Author's research more valuable.	Thank you for your insightful comment. We confirm that the experiment was conducted once, as part of an MS thesis project. Due to the limited time frame available within the academic schedule, it was not possible to repeat the experiment across multiple seasons. However, all treatments were arranged in a randomized complete block design (RCBD) with three replications to ensure the reliability and statistical validity of the results. As per your suggestion, we have now included this information in the Abstract to enhance the clarity and value of our research.
3. **Introduction**	Line 32: According to the conventions of biological nomenclature, taxonomic ranks above genus—such as

Line 32, Please make Solanaceae italic. line 35: MT, please write out the first time metric ton and then use the abbreviation in parentheses (MT), later authors can use only the abbreviation.<br>lines 42-44: Please rephrase the sentence and note that there are several Fusarium species which can infect brinjal, but the most important of these species is the most devastating, Fusarium oxysporum f. sp. melongenae. lines 46-47: please add that Fusarium oxysporum survives in the soil with its chlamydospores.<br>line 48: measures (use the plural of measure) line 54: Bio-control agents are required to go through several official test in Europe, USA and Australia among other countries. Instead of bio-control measure I would use the terms "organic soil additives" or "organic byproducts". I would use use "poultry manure" instead of "poultry refuse".<br>line 56: please explain what is SMC line 57: please explain what is SMS (like in the Abstract the Authors did) line 60: use plural of earthworm	family names like *Solanaceae*—are not italicized. Therefore, we have retained the standard formatting for the family name in the manuscript.<br>Lines 42-44: As recommended, we have rephrased the sentence to clarify.<br>Lines 46-47: As recommended, the information is added.<br>Line 48: Changed from measure to measures.<br>Line 54: Changed according to the recommendations.<br>Line 56: I It was a typographical error - "SMC" should be corrected to "SMS," which stands for Spent Mushroom Substrates. The necessary correction has been made in the revised manuscript.<br>Line 57: SMS = Spent Mushroom Substrates (Treatment 1-T_1)<br>Line 60: Changed from earthworm to earthworms.
4. **Materials used**<br>lines 74-76: delete it, it is redundant. line 106: I feel that 6 plants are not enough to make the statistics reliable. The more plants one has in a treatment the more reliable the results are.<br>line 123: add "moistened" blotting paper. line 124: add "in a humid chamber". line 132: what was the volume of the pots used? line 136: how were the roots damaged? Did the authors tried just dipping the seedlings in the spore suspension?	Lines 74-76: The lines have been deleted.<br>Line 106: Thank you for your observation. In this experiment, each plot contained 6 plants, and there were 3 replications per treatment, resulting in a total of 18 plants for each treatment. We agree that a larger number of plants could contribute to more accurate and robust results. However, due to limitations in available land resources within the university campus, it was not feasible to increase the plant population in this study.<br>Line 123: The information is added.<br>Line 124: The information is added.<br>Line 132: The volume of the pots used is 15 liters. The information is updated in the manuscript.<br>Line 36: Thank you for your thoughtful comment. We apologize for the lack of clarity in the original description. To enhance pathogen infection efficiency during the pathogenicity test, the roots of 30-day-old seedlings were **intentionally wounded** by gently trimming them with sterilized scissors before being immersed in the spore suspension. This step was taken to facilitate infection by simulating natural root injury. The revised sentence in the manuscript now clarifies this procedure.

5. **Results**<br>lines 149-158: In scientific publishing it is expected that the Authors carry out pathogen identification with 2 different methods, one method should result in a molecular evidence, like sequencing a genetic barcode region or housekeeping gene. Without molecular evidence one cannot reliably state that a pathogen is identified and causes the disease. For Fusarium species the tef1 gene is used for identification. This gene should be used to identify the pathogen before and after carrying out the Koch's postulates. Scientific rigor also requires that the entire trial is repeated at least twice in consecutive growing seasons or at different locations.	Lines 149-158: Thank you for your insightful recommendations. We fully agree that molecular identification, particularly using PCR amplification and sequencing of housekeeping genes such as *tef1*, is a more reliable approach for distinguishing closely related *Fusarium* species infecting *Solanum melongena*. However, the primary objective of this study was centered on disease management strategies rather than detailed taxonomic classification. As a result, molecular characterization was not performed at the time of the experiment.<br>Furthermore, the experiment was conducted during the 2021–2022 period as part of an MS thesis project, and the fungal cultures are no longer preserved, making retrospective molecular identification unfeasible. In light of this limitation, and to maintain scientific accuracy, we have revised the manuscript to refer to the causal agent as *Fusarium* sp. rather than *Fusarium oxysporum* f. sp. *melongenae*.<br>Regarding the experimental repetition, we acknowledge that repeating the trial across multiple seasons or locations would enhance the scientific rigor of the findings. However, due to time and resource constraints inherent to the academic program, the trial was conducted only once. Despite this, the experiment was designed using a randomized complete block design (RCBD) with three replications to ensure internal validity. Additionally, our results are consistent with previous studies conducted in various regions, suggesting that the findings are reliable and applicable to similar agroecological contexts.<br>We consider this work a preliminary investigation and plan to conduct further studies incorporating molecular diagnostics and multi-season/location trials to strengthen and validate the findings.
6. What I'm missing is quality photographs of the wilted plants to show the effectiveness of the different treatments. Because all the plants were potted it would be easy for the Authors to collect 1 demonstrative example from each treatment, put them side by side and take a picture. This visualization would greatly enhance the value of the publication. There are no symptom photographs, just tables graphs and an image from a culture and macro-conidia.	Thank you for your thoughtful suggestion. We would like to clarify that the main experiment was conducted under field conditions following a randomized complete block design (RCBD), not in potted plants. Only Koch's postulates were carried out in pots to confirm the pathogenicity of the isolated organism. Therefore, it was not feasible to arrange all treated plants side by side for comparative photography as suggested.<br>Additionally, all treatments in the field trial produced similar wilt symptoms, and the variation was primarily observed in terms of disease severity rather than symptom type. As a result, displaying multiple images of similar symptoms would be redundant. However, in response to your comment, we have now

	included a representative photograph [Fig. 1] of a wilted brinjal plant to illustrate the typical disease symptoms observed in the study. We hope this visual addition enhances the clarity and impact of our findings.
7. I also would expect tho have this tiral setup repeated at a different location or different year, because weather changes from year to year and from location to location. In science one cannot draw reliable conclusion from one trial from one location.	Thank you once again for your valuable observation. As mentioned in our previous response, we fully acknowledge the importance of repeating trials across different years or locations to enhance the reliability and generalizability of the findings. However, this study was conducted as part of an MS thesis project within a limited academic timeframe and available resources, which restricted us to a single season and location. Despite this limitation, the experiment was carefully designed using a randomized complete block design (RCBD) with three replications to ensure statistical validity. Additionally, the outcomes of our study are consistent with similar research reported in other regions. We consider this work a preliminary investigation, and future studies are planned to include multi-location and multi-season trials to validate and strengthen these findings further.
8. This trial has shown great potential for the use of bio-organic matters as soil amendments to combat Fusarium wilt of Solanum melongena. However it appears that the results and conclusions are not supported well enough with data in this current form of the MS, so I suggest a major revision and expansion of the results section from data from a different location OR from at least 2 growing seasons.	Thank you for your continued engagement and thoughtful recommendation. We understand the importance of validating results through multi-season or multi-location trials to strengthen scientific conclusions. However, as previously mentioned, this study was conducted during the 2021–2022 period as part of an MS thesis project, and due to the constraints of time and resources, it was not possible to repeat the experiment.<br>That said, we have made a conscious effort to structure our discussion clearly and meaningfully. To better highlight and support our findings, we revised the discussion section by dividing it into three distinct thematic subheadings, each aligned with our specific research objectives and supported by relevant literature. Similarly, the conclusion has been carefully framed to reflect the core findings and scope of our study.<br>We kindly request that, if the reviewer feels any specific data, interpretations, or clarifications are missing in the results, discussion, or conclusion sections, those points be specified with reference to the relevant lines. This would allow us to make targeted improvements in line with your expectations.<br>Although we are unable to repeat this particular experiment due to its completion in 2021–2022, we highly value your suggestions and will certainly

	incorporate them in the design and implementation of future research projects.
**Reviewer #2**	
1. The assessment of microbial diversity should be expanded by including detailed methods or data on soil microbial communities to strengthen related claims.	Thank you for your thoughtful comment. We agree that assessing soil microbial diversity can provide valuable insights; however, the primary objective of this study was specifically focused on the management of *Fusarium* wilt in brinjal using organic amendments, rather than characterizing the broader microbial community in the soil.<br>While we acknowledge that including microbial diversity data could enrich the study, such analyses were beyond the defined scope and resources of this project. In scientific research, especially in focused studies such as this, it is important to remain aligned with the specific objectives to maintain clarity and coherence. Comprehensive assessments of soil microbial communities are certainly valuable and would be more appropriate for a separate study or a broader review, such as in a book chapter.<br>That said, we highly appreciate your suggestion and will consider incorporating microbial community analysis in future research that aims to explore the wider ecological impacts of organic amendments.
2. A control treatment with a chemical fungicide should be included or discussed to provide a benchmark for comparing the efficacy of bio-organic amendments.	Thank you for your valuable suggestion. In this study, our primary objective was to evaluate and compare the effectiveness of different bio-organic amendments in managing *Fusarium* wilt of brinjal, with a focus on identifying sustainable alternatives to chemical control. Therefore, we included several organic treatments along with an untreated control, but did not include a chemical fungicide treatment, as a comparison between organic and chemical management strategies was beyond the scope of this research.<br>We fully acknowledge that chemical fungicides may offer higher short-term efficacy; however, the emphasis of our work was on exploring environmentally friendly and sustainable solutions that minimize negative impacts on human health and the ecosystem. Future studies may incorporate chemical treatments as benchmarks to provide a broader comparison between conventional and sustainable management approaches.
3. The characteristics of the biochar used, such as source material and pyrolysis conditions, should be detailed to enhance reproducibility and understanding of treatment effects.	Thank you for your valuable suggestion. We acknowledge the importance of providing detailed information about the biochar to enhance the reproducibility and scientific understanding of its

	effects. The biochar used in this study was prepared from rice husk under controlled pyrolysis conditions. Additional details, including the source material and pyrolysis parameters, have now been added to the Materials and Methods section to improve clarity.
4. The variability observed in disease incidence and yield data, indicated by high coefficients of variation, should be addressed with discussion of potential causes and management approaches.	We thank the reviewer for pointing out the variability observed in disease incidence and yield data. In response, we have now added a dedicated subsection in the discussion titled **"Treatment-Based Variability in Disease suppression and yield response"** to address this issue in detail. We hope this addition addresses the concern adequately.
5. Soil physicochemical analyses, including nutrient status and pH before and after treatments, should be provided to support conclusions about soil health improvements.	We appreciate the reviewer's valuable suggestion. While we agree that soil physicochemical properties such as nutrient status and pH are important indicators of soil health, our study was primarily focused on the **pathological management of Fusarium wilt in brinjal**, rather than a comprehensive assessment of agronomic improvements. Due to the specific scope of our research objectives and limitations in resources, we concentrated on evaluating the disease suppression efficacy of various bio-organic treatments. However, we acknowledge that soil health plays a crucial role in disease dynamics, and we plan to incorporate detailed soil physicochemical analyses in our future research to further validate and support such conclusions.
6. The long-term implications of the treatments on disease suppression and soil health should be discussed, considering the persistent nature of Fusarium oxysporum.	We thank the reviewer for highlighting this important aspect. We fully agree that the long-term implications of the bio-organic treatments are critical, especially considering the persistent nature of *Fusarium oxysporum* in the soil. While our current experiment was conducted over a single season due to resource and time limitations, we have now expanded the discussion to include the potential long-term benefits of continued application of these amendments, citing relevant literature to support this perspective. We believe this addition enhances the overall understanding of the sustainability and broader impact of bio-organic disease management strategies. The new content can be found in the revised discussion section under the subheading "Long-term implications of bio-organic amendments."
7. The quality and clarity of figures and legends should be improved by adding detailed descriptions and explaining all symbols and abbreviations.	Thank you for your valuable comment. In accordance with the PLOS One journal guidelines, figure captions are provided after the paragraphs where the respective figures are first mentioned, rather than embedded within the figure files. Therefore, the figure files themselves contain only the figure numbers. However, we fully acknowledge the importance of figure clarity and descriptive legends. If there are

	specific figures or symbols/abbreviations that you feel require further clarification or additional information in the legends, we would be grateful if you could kindly specify them. We will be happy to revise and improve those figure legends accordingly to enhance clarity and reader understanding.
8. Minor grammatical and typographical errors should be corrected through careful proofreading for polished presentation.	Thank you for pointing this out. The manuscript has been carefully proofread, and all identified grammatical and typographical errors have been corrected to ensure a polished and professional presentation.
9. As the results demonstrate that the treatment with biochar outperformed the other treatments in different characters. The authors must give credit to and cite the recent literature on the application biochar for disease resistance in different crops, for instance,, the review by Arshad (2024) (10.55627/pbiotech.002.01.0972), Arshad et al. (2020, https://doi.org/10.17957/IJAB/15.1522) and (2021, https://doi.org/10.1007/s10343-021-00580-4) are highly relevant to this research and should be incorporated in the discussion.	Thank you for your valuable suggestion. The 2nd Reference is aligned with our objectives and added in the discussion as reference number [25].
10. Similarly, recently, Fayyaz et al. have reported different biochars for nematode management in tomato (10.21162/PAKJAS/24.30). These reports must be cited in this review of literature.	Thank you for bringing this paper to our attention. The study by Fayyaz et al. focuses on the use of biochar for nematode management in tomato, whereas our study specifically targets Fusarium wilt, caused by the fungal pathogen *Fusarium oxysporum*. Since nematodes and fungi belong to entirely different biological kingdoms and have distinct infection mechanisms and management approaches, we believe the direct relevance of that study to our fungal disease-focused research is limited. However, if you feel the general approach or mechanisms described in the study could enrich our discussion, we would be happy to reconsider its inclusion with your further guidance.
**Response to Reviewer #3**	
1. The abstract provides a broad summary of the study's intent but lacks numerical data and statistical validation of findings. It overstates the results without sufficient context or limitations.	Thank you for your valuable observation. In response, we have revised the abstract to include specific numerical data on disease incidence (at 25, 45, and 65 DAT) for both the untreated control (T_0) and the most effective treatments (T_5 and T_6). We have also clarified that the differences were statistically significant to provide stronger validation of the findings. These revisions ensure a more balanced and evidence-based summary of the results while avoiding overstatements.
2. The introduction is overly general and lacks citations from recent, high-impact literature. No clear hypothesis is defined. Rationale behind the	Thank you for your insightful comments. We respectfully request that you kindly specify the areas that require improvement in a line-by-line manner, as

selection of bio-organic treatments is poorly developed.	was done by Reviewer #1, to help us better understand the concerns regarding the introduction. The citations we included are both recent and relevant to the scope of our study. If there are more recent or high-impact studies that you believe should be included, we would greatly appreciate it if you could provide their DOI or publication links.<br>Additionally, we are not aware of any specific PLOS One guideline that requires a minimum impact factor for cited literature. If such a guideline exists, we kindly ask you to share it with us. Furthermore, if any of the references used in our manuscript fall below the acceptable impact threshold, we would be grateful if you could identify them and recommend more suitable, high-impact alternatives relevant to our study topic.<br>We are fully committed to enhancing the quality of the manuscript and will incorporate any suggested citations that improve the clarity, depth, or scientific rigor of the introduction.
3. In methodology experimental design lacks clarity like randomization, replication, and plot size are not clearly mentioned.	Thank you for your observation. We would like to clarify that the details regarding randomization, replication, and plot size are already provided under the subheading **“Experimental design”** in the methodology. Specifically, we stated that the study was laid out in a Randomized Complete Block Design (RCBD) with eight treatments and three replications. The experimental field was divided into three blocks, each containing eight plots, resulting in a total of 24 plots. The size of each plot was 2.5 × 1.8 m, with 0.75 m spacing between blocks and 0.50 m spacing between plots. Each plot had six plants, with a plant-to-plant distance of 75 cm. A field layout is added now as a supporting file **(S1 Appendix).**<br>We respectfully request you to kindly review this section again, and if you still feel that any additional information is required for clarity, please indicate the specific points or lines so that we can address them accordingly.
4. No information on soil physico-chemical properties before treatment application is presented.	The information is added in the methodology under the subheading of “Baseline soil physicochemical properties”.
5. The absence of details on Fusarium wilt inoculum preparation and application undermines the disease pressure standardization and data collection frequency as well as disease rating scale is not sufficiently described.	Disease incidence assessment does not require a rating scale, as it is calculated based on the percentage of infected plants. A rating scale is only essential for disease severity measurement. In our study, disease incidence was determined using an equation that relies on counting the number of infected plants, and this equation was already provided in the methodology section. Details of *Fusarium* wilt inoculum preparation and application have now been

	incorporated into the methodology to address the concern regarding disease pressure standardization.
6. The discussion lacks depth and critical analysis. Several repetitive and descriptive statements rather than analytical in the discussion. Also add discussion of limitations, which is essential for transparency. Similarly, author failed to explore the mechanism behind the suppression of Fusarium	In the discussion, several additional points have been included to enhance its depth and clarity. Since the research was conducted during 2021–22, please let us know if there are any specific aspects or recent developments you would like us to incorporate at this stage.
7. The manuscript contains multiple typographical errors, grammatical mistakes, and inconsistent formatting. Scientific names are inconsistently italicized. Units and abbreviations are not standardized throughout.	We have thoroughly reviewed the manuscript and carefully corrected all typographical errors, grammatical mistakes, and formatting inconsistencies. Scientific names have been standardized with proper italicization, and units and abbreviations have been consistently formatted throughout the text.

We look forward to your further evaluation of our revised manuscript.

Sincerely,

Raihan Ferdous

Corresponding author
