# Supplementary material for: Optimizing brinjal (Solanum melongena L.) health and yield through bio-organic amendments against Fusarium wilt
Source: PLoS One. 2025 Oct 9;20(10):e0325345. doi: 10.1371/journal.pone.0325345 (PMC12510524; doi:10.1371/journal.pone.0325345)
Supplement: Attachment — Submitted filename: Response_to_Reviewers_auresp_2.pdf [file pone.0325345.s004.pdf]

Date: 07-09-2025

Academic Editor,
PLOS ONE

Dear Ravinder Kumar,

The author would like to thank you and the reviewers for your thoughtful and constructive feedback on our manuscript [PONE-D-25-25430R1]. The author greatly appreciates the time and effort put into reviewing our work, and we have carefully considered each point raised. In response, the author has made the necessary revisions and clarifications throughout the manuscript to address all concerns.

Enclosed is a table detailing each comment made by the reviewers, alongside author responses and the corresponding revisions. The author believes these changes have strengthened the manuscript, and we hope that it now meets the journal's standards.

**Reviewer #1**	**Response by Author**
Line 155: "@ 200ml/cft" - I'd kindly ask the authors not to use @, instead write out "at" and instead of using cft (= cubic feet?), I strongly recommend to use SI units as recommended in the Journal's submission guidelines.	Thank you for the valuable suggestion. We have revised the text accordingly. The symbol "@" has been replaced with "at," and the non-SI unit "cft" (cubic feet) has been converted into SI units as per the journal's submission guidelines. Specifically, "200 ml/cft" has been expressed as **7.0 L/m³** in the revised manuscript (Line 155).
In line 160-161 the authors state the used Fusarium oxysporum f. sp. melongenae, but in their response they state the pathogen was identified my classical methods, and the original cultures are not available for further molecular testing. Identifying a pathogen by only classical methods does not meet scientific rigor not allows reproducibility.	We sincerely appreciate the reviewer's insightful comment. We acknowledge the limitation that the pathogen was identified only by cultural and morphological characteristics, and molecular confirmation could not be performed as the original cultures are no longer available. To maintain scientific rigor and ensure reproducibility, we have revised the pathogen name from ***Fusarium oxysporum* f. sp. *melongenae* to *Fusarium* sp**. in the manuscript (Lines 160–161). Through cultural and morphological methods, we are confident in confirming the genus, but not the species.
Line 169: The authors should state what was the volume of the conidial suspension. It is also not clear weather the roots of the seedlings were washed from soil or they carried out the dip method with soil. If it was carried out with soil the soil absorbs part of the conidial suspension it was dipped in, this is why the volume of the conidium suspension is important. More absorbed volume menas more inoculum per plant.	We have revised the manuscript to clearly state the volume of the conidial suspension and clarified that the roots were washed before immersion. The corrected description is now included in Line 169: **"The wounded roots were carefully washed to remove adhering soil particles and then immersed in 20 ml of a conidial suspension of *Fusarium* sp. (10^6 conidia/ml) for 10 minutes."**
line 279-282: "The authors reported that the application of SMS increased the diversity of	We appreciate the reviewer's careful observation. We agree with the comment that this statement is not

fungi, including Tremellomycetes and Pezizomycetes for the SMS additive, while the levels of Mortierellomycetes, Pezizomycetes, and Leotiomycetes increased after the addition of poultry manure." This sentence does not make sense and should be deleted. The authors did not test the microbial community of the plots before or after the trial, and have no data to substantiate this statement.	supported by our data, as we did not analyze the microbial community before or after the trial. Accordingly, we have deleted the sentence in Lines 279–282 from the revised manuscript.
**Reviewer #2**	
application biochar for disease resistance in different crops, for instance,, the review by Arshad (2024) (10.55627/pbiotech.002.01.0972) where the author has demonstrated the role and mechanisms of biochar for enhancing resistance to pathogens and insects.	Thank you for the constructive suggestion. We have revised the introduction to include the application of biochar for disease resistance in different crops, as highlighted in the review by Arshad (2024), citation [13]. The following sentence has been added with the appropriate citation (Line 72-76): **"Biochar acts as a biocontrol agent against various plant pathogens and insect pests by modulating plant physiological functions (such as the activation of defense-related genes), altering rhizosphere chemistry, enhancing beneficial microbial communities, and releasing allelopathic compounds that suppress pathogen development [13]."** However, this citation is also mentioned in the discussion part (Line 296).

We look forward to your further evaluation of our revised manuscript.

Sincerely,

Raihan Ferdous

Corresponding author
